# Antiseptics’ Concentration, Combination, and Exposure Time on Bacterial and Fungal Biofilm Eradication

**DOI:** 10.1016/j.artd.2024.101468

**Published:** 2024-07-23

**Authors:** Emanuela Roscetto, Donato Di Gennaro, Tiziana Ascione, Umberto Galdiero, Martina Aversa, Enrico Festa, Maria Rosaria Catania, Giovanni Balato

**Affiliations:** a Department Molecular Medicine and Medical Biotechnology, “Federico II” University, Naples, Italy; b Section of Orthopaedic Surgery, Department of Public Health, “Federico II” University, Naples, Italy; c Department of Medicine, Service of Infectious Disease, Cardarelli Hospital Naples, Naples, Italy

**Keywords:** Biofilm, Periprosthetic joint infection, PJI, Hydrogen peroxide, Antiseptics

## Abstract

**Background:**

This study aims to assess the activity of solutions containing povidone-iodine (PI) and hydrogen peroxide (H_2_O_2_) alone or combined on the biofilm of microbial species in the contest of periprosthetic joint infection (PJI).

**Methods:**

Different antiseptic solutions were tested on 2-day-old biofilms of Gram-positive and Gram-negative bacteria and fungi at 1 and 3 minutes of exposure. The efficacy of these solutions was evaluated by measuring the biofilm metabolic activity by methoxynitrosulfophenyl-tetrazolium carboxanilide (XTT) reduction assay. The anti-biofilm effect of 5% PI and 0.3% PI + 0.5% H_2_O_2_ was tested on a 5-day-old biofilm using colony-forming unit counts and an XTT reduction assay.

**Results:**

PI and H_2_O_2_ solutions showed concentration-dependent anti-biofilm activity except for *E. faecalis*. PI at 5% was the most active solution against the 2-day-old biofilm of all test microorganisms. The 0.3% PI + 0.5% H₂O₂ solution had a significant effect only at 3 minutes. The 5% PI and 0.3% PI + 0.5% H₂O₂ effect was evaluated on 5-day-old biofilms. PI at 5% produced a significant reduction in metabolic activity at both 1 and 3 minutes; 0.3% PI + 0.5% H₂O₂ caused a significant activity against all Gram-positive strains after 3 minutes, with a greater metabolic activity reduction than 5% PI.

**Conclusions:**

In the case of PJI caused by Gram-positive bacteria, 0.3% PI + 0.5% H₂O₂ could be used for wound irrigation for 3 minutes of exposure. In the case of PJI with a different etiological agent or PJI with an unknown etiology, it is advisable to use 5% PI for 1 minute of exposure.

## Introduction

The rise in biofilm-related infections has dramatically decreased the success rate of surgical procedures, increasing patient morbidity and mortality [[1], [2], [3]]. In orthopaedic surgery, prosthetic joints and osteosynthesis devices represent the ideal setting for biofilm development [[4], [5], [6], [7], [8], [9], [10], [11]]. Indeed, the treatment of periprosthetic joint infection (PJI) and fracture-related infections could include hardware removal, accurate debridement, and antibiotic therapy with an eradication rate that ranges between 63% and 89% for fracture-related infection and between 80% and 88% for 2-stage revision arthroplasties [[12], [13], [14], [15], [16], [17], [18], [19], [20], [21]]. The presence of a foreign body facilitates bacterial adhesion and allows biofilm formation. A strategy to reduce the bacterial load and eradicate the biofilm is to use antiseptic agents at the surgical site. Different antiseptic solutions are available, but only a few clinical studies are proposed to evaluate their role in eradicating biofilm infection. For surgical site irrigation, 0.3% povidone-iodine (PI) is recommended, thanks to its safety profile, activity, and low cost [[22], [23], [24]]. Often associated with PI, hydrogen peroxide (H_2_O_2_) is another widely used antiseptic, especially in spinal surgery [25,26]. Recently, a new irrigation solution consisting of 0.3% PI and 0.5% H_2_O_2_ was proposed as an adjuvant for treating PJI [27]. The rationale for using combined solutions of PI and H_2_O_2_ is linked to their mechanism of action. The microbicidal activity of iodine results from its ability to oxidize the sufhydryl and hydroxyl groups of proteins, leading to the death of the microbe. H_2_O_2_ also binds and oxidizes the sufridilic groups [28]. Mixing low concentrations of PI and H_2_O_2_, PI will attack some of the thiol groups, and H_2_O_2_ can oxidize the other portion. The antibiofilm activity of these antiseptic solutions at different concentrations on various species of bacteria and fungi is not yet well described. So, this study aims to compare the activity of 6 solutions of PI and H_2_O_2,_ alone or in combination, to eradicate mature biofilm of methicillin-resistant *Staphylococcus aureus* (MRSA), *S. epidermidis, E. faecalis, P. aeruginosa,* and *C. albicans.* In this study, we asked: i) Which solution performs best in eradicating bacteria embedded in a mature biofilm? and, ii) What minimum irrigation time is required to eradicate mature biofilm?

## Material and methods

### Antiseptic solutions

All solutions were prepared starting at 10% PI and 3% H_2_O_2_ (Table 1). We proceeded to the dilution with 0.9% NaCl and then to the combination to obtain the final solutions. The solutions were prepared under sterile conditions and filtered using a 0.2 μm filter.

**Table 1:** Antiseptic solutions.

Solutions tested	Materials^a^
PI 0.3%/H_2_O_2_ 0.5%	15 mL PI 10%/83 mL H_2_O_2_ 3%/402 mL NaCl 0.9%
H_2_O_2_ 0.5%	417 mL NaCl 0.9%/83 mL H_2_O_2_ 3%
PI 0.3%	485 mL NaCl 0.9%/15 mL PI 10%
PI 5%/H_2_O_2_ 1.5%	250 mL PI 10%/250 mL H_2_O_2_ 3%
H_2_O_2_ 1.5%	250 mL NaCl 0.9%/250 mL H_2_O_2_ 3%
PI 5%	250 mL NaCl 0.9%/250 mL PI 10%

a Materials shown are intended for a volume of 500 mL.

### Tested strains and culture conditions

This study tested 5 reference strains: *Pseudomonas aeruginosa* PAO1, *Staphylococcus epidermidis* American Type Culture Collection (ATCC) 1228, *Enterococcus faecalis* ATCC 29212, methicillin-resistant *Staphylococcus aureus* ATCC 43300, and azole-resistant *Candida albicans* ATCC 10231. All strains were stored as 15% (v/v) glycerol stocks at −80°C. Before each experiment, bacterial cells were sub-cultured from the stocks onto tryptic soy agar (and/or blood tryptic soy agar; Becton Dickinson, Franklin Lakes, NJ) plates at 37°C for 24 hours, while Candida cells were sub-cultured onto Sabouraud dextrose agar (Becton Dickinson) at 37°C for 24-48 hours. Identification was performed by biochemical characterization using the Vitek2 (Biomerieux, Mercy-l’Etoile, France) and confirmed by MS MALDI-TOF (Bruker Daltonics, Bremen, Germany).

### Formation of microbial biofilm and 2,3-bis(2-methoxy-4-nitro-5-sulfo-phenyl)-2H-tetrazolium-5-carboxanilide (XTT) assay

Biofilms of the test strains were grown for 2 and 5 days in flat-bottomed 96-well microtiter plates. First, 0,5 McFarland cell suspensions in Brain Heart Infusion for bacterial strains (1.5-5 × 10^8^ colony-forming unit [CFU]/mL) and Sabouraud dextrose broth for *Candida* strain (4 × 10^6^ CFU/mL per well) were prepared. Aliquots (200 μL) of 1:100 dilutions in fresh media supplemented with 1% glucose were added to each well in triplicate. A negative control was prepared by inoculating 200 μL of a cell suspension inactivated by boiling. The plates were then incubated at 37°C under static conditions. Fresh media was exchanged every 24 hours. After 2 and 5 days, the supernatants were gently aspirated to remove floating cells, and the plates were washed twice with sterile phosphate-buffered saline (PBS, pH 7.2). The wells were exposed in triplicate to 200 μL of each test solution for 1 minute and 3 minutes. Treatment-free wells were included as positive controls. After treatment, the medium was aspirated, the wells were thoroughly washed twice with PBS, and an methoxynitrosulfophenyl-tetrazolium carboxanilide (XTT) reduction assay was performed as described below [29]. The XTT-reduction assay has been used to quantitatively measure microbial metabolic activity, growth, and response to antimicrobial treatments [29,30]. An XTT cell proliferation Kit II was purchased from Roche Diagnostics. Two hundred microliters of XTT solution were added to each well, and the plate was incubated in the dark for 3 hours at 37°C. Changes in the absorbance of XTT were measured spectrophotometrically at 490 nm using a microtitre plate reader (Biorad). Viability ratios were computed for each well concerning their relative controls.

### Colony-forming units (CFU) assay

After the treatment, the efficacy of tested solutions on biofilms after 5 days was determined by counting CFUs. The wells were gently washed twice with PBS to remove the antiseptics. Aliquots of 200 μL of dithiothreitol 0.1% was added to each well. The microplate was sonicated in an ultrasonic bath (T460, Elma Gene Probe) for 30 minutes at 35 kHz. Serial dilutions of each well were plated onto Mueller-Hinton agar plates and incubated at 37°C for 24 hours. CFU log_10_ reduction was calculated by comparing the CFUs of each well to the CFU value of control (without treatment) for each strain. The assay was performed in triplicate.

### Statistical analysis

Continuous data were summarized using mean and standard deviation. The antibiofilm activity for each exposure time was calculated and expressed as optical density. The inhibition of biofilm metabolic activity was also evaluated and expressed as the mean percentages ± standard deviation (SD). Data were analyzed using a one-way analysis of variance, followed by Tukey’s post hoc test. This test was used to compare (i) the activity of a single solution at a different time of exposure (1 and 3 minutes) and (ii) the activity of different solutions at the same time of exposure. Furthermore, for statistically significant differences, we performed a post hoc test as a subgroup analysis to highlight the differences between groups. Colony counts were reported as means and standard deviations and compared using an independent *t*-test. The level of significance was set at *P* < .05. The IBM SPSS Statistics for Windows, Version 23.0 (IBM Corp., Armonk, NY) was used for database construction and statistical analysis.

## Results

### Anti-biofilm activity against 2-day-old biofilm: effect on the metabolic activity

**Figure 1:**
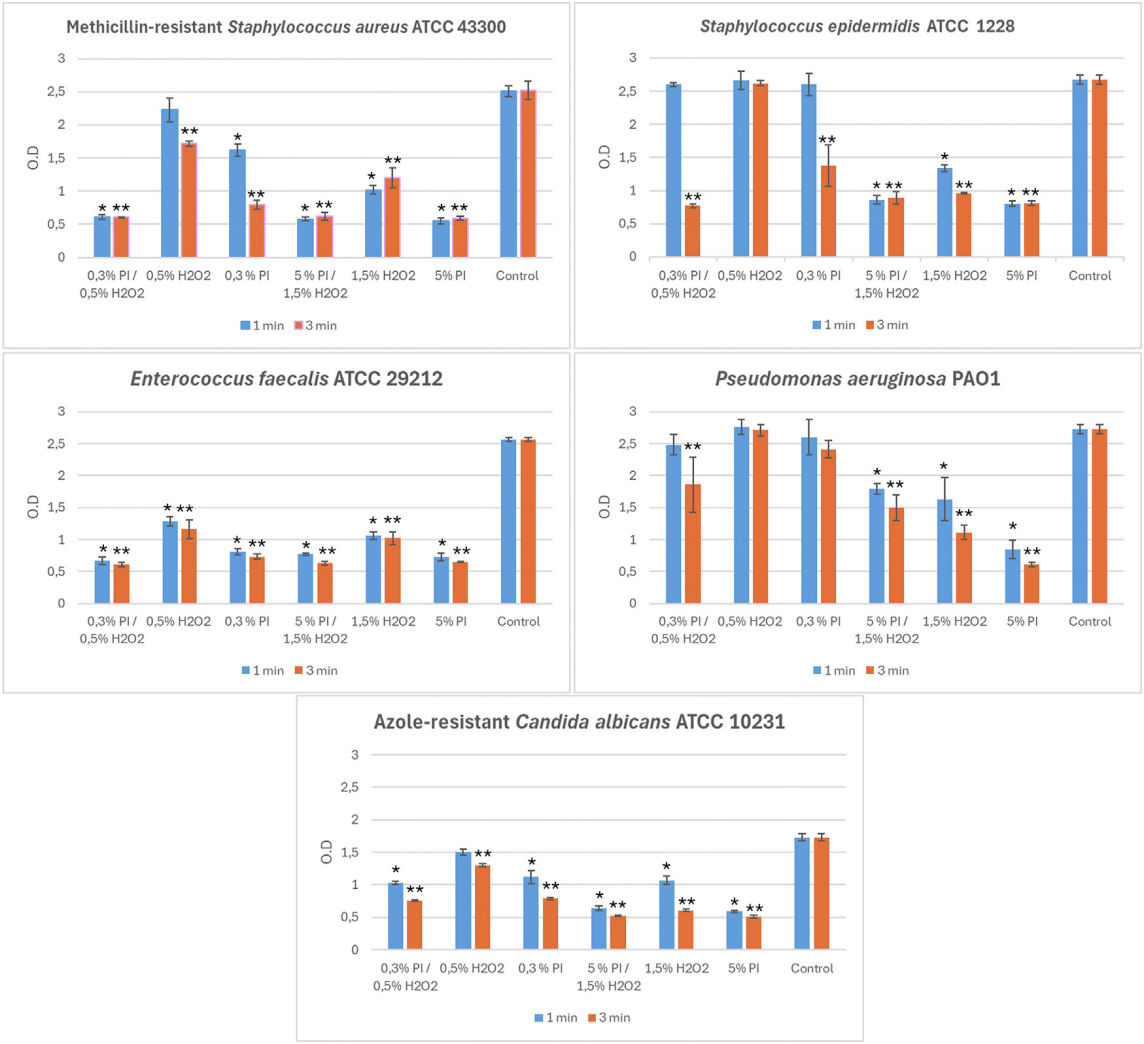
Mean values of the optical density (±standard deviation) of the 2-day biofilm of the strains under examination following treatment at 1 and 3 minutes with the antiseptic solutions. ∗ Statistical differences with control at 1 minute of exposure (*P* < .001). ∗∗ Statistical differences with control at 3 minutes of exposure (*P* < .001).

Figure 1 shows the activity of different antiseptic solutions on the metabolic activity of 2-day-old biofilm of the different test strains. Compared to the untreated control, PI produced a statistically significant decrease in the metabolic activity of biofilm of all test strains only at the highest concentration (5%) at both exposure times (1 and 3 minutes). At 1 minute of exposure, 0.3% PI was active only against the biofilm of *S. aureus*, *E. faecalis,* and *C. albicans*; at 3 minutes, it was active also against S. epidermidis but not against P. aeruginosa 48-hour biofilm. At a concentration of 1.5%, H₂O₂ significantly affected the metabolic activity of the biofilm of the different species at both treatment times, but the effect increased with the time of exposure for *S. epidermidis, P. aeruginosa,* and *C. albicans*. The less concentrated solution (0.5% H₂O₂) was active only against *E. faecalis* biofilm after 1 minute; at 3 minutes of exposure, the solution was active also against MRSA and *C. albicans* biofilms. The combination of PI and H₂O₂ at the lowest concentrations significantly reduced the metabolic activity of all microbial biofilms only at the longest exposure time (3 minutes). In fact, at 1 minute of exposure, the activity of 0.5% PI and 0.3% H₂O₂ on *S. epidermidis* and *P. aeruginosa* was not statistically different compared to controls. Their combination at the highest concentrations significantly affected all test strains at both 1 and 3 minutes of treatment (Tables reporting the anti-biofilm activity of test solutions determined by the XTT assay are provided in Appendix 1).

### Anti-biofilm activity of 5% PI and 0.3% PI + 0.5% H₂O₂ combination against 5-day-old biofilm: effect on the metabolic activity

**Figure 2:**
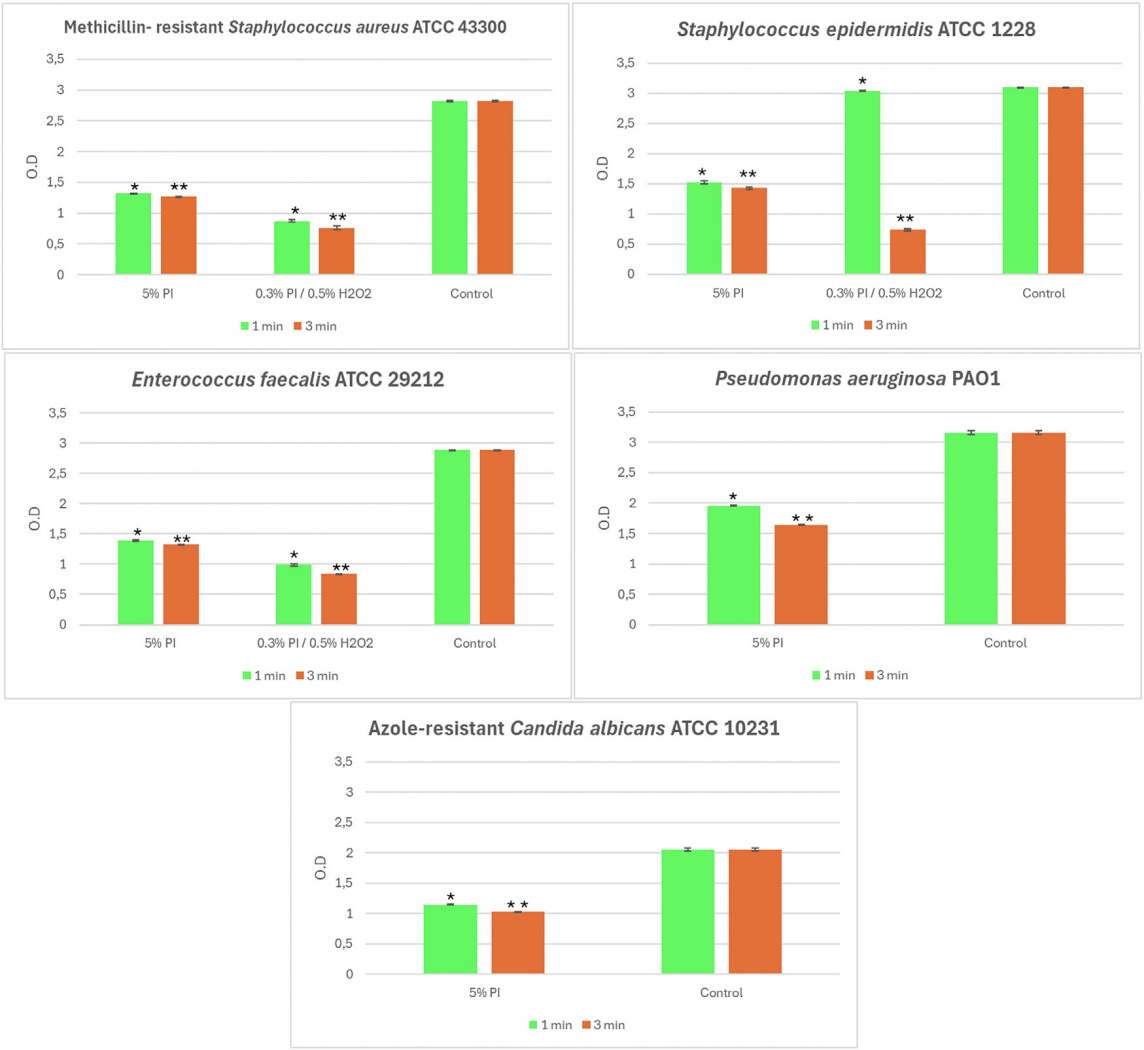
Mean values of the optical density (±standard deviation) of the 5-day biofilm of the strains under examination following treatment at 1 and 3 minutes with the antiseptic solutions. ∗ Statistical differences with control at 1 minute of exposure (*P* < .001). ∗∗ Statistical differences with control at 3 minutes of exposure (*P* < .001).

Based on previous experiments on 2-day-old biofilms, the activity of 5% PI and 0.3% PI + 0.5% H₂O₂ combination was also evaluated on 5-day-old mature biofilms (Fig. 2). The 5% PI solution was tested against mature biofilm of all test strains, showing a significant (*P* < .001) reduction in metabolic activity at both exposure times. The combined solution of 0.3% PI + 0.5% H₂O₂ was tested on mature biofilm of the Gram-positive strains, as its anti-biofilm activity was comparable to that of 5% PI against the 2-day biofilm of these bacteria at the longest time of treatment. After 1 minute of exposure, the combined solution did not produce a significant reduction in the metabolic activity of *S. epidermidis* mature biofilm. After 3 minutes of exposure, it caused a significant reduction in the biofilm of all 3 strains, and its anti-biofilm activity was significantly greater than 5% PI solution (Tables reporting the anti-biofilm activity of test solutions determined by XTT assay are provided in Appendix 1).

### Anti-biofilm activity of 5% PI and 0.3% PI + 0.5% H₂O₂ combination against 5-day-old biofilm: effect on the colony-forming unit counts

The activity of 5% PI and 0.3% PI + 0.5% H₂O₂ combination against mature biofilm was also evaluated by counting colony-forming units, and the results are shown in Table 2 and Table 3. Each solution was tested only against the strains on which it was effective in previous experiments. For Gram-positive strains, 5% PI solution caused a reduction in CFU of at least 93% compared to the control after 1 minute exposure, and of at least 99.4% after 3 minutes. The effect on other species was less notable: only the longer exposure time caused a reduction in CFU of 78% and 75% for *P. aeruginosa* and *C. albicans*, respectively. After 1 minute exposure, 0.3% PI + 0.5% H₂O₂ combined solution had a greater effect than 5% PI on MRSA (*P* = .018) and *E. faecalis* biofilms (*P* < .001). Regarding *S. epidermidis*, combined solution had a weak effect compared to the control (CFU reduction of 13.5%). However, after 3 minutes of treatment, the effect was statistically superior to that of 5% PI against all Gram-positive strains (*P* < .001) (Table 3).

**Table 2:** Effect on the colony-forming unit counts of antiseptic solutions.

Bacterial strain	PI 0.3%/H2O2 0.5%			PI 5%		
	1 min	3 min	*P* value	1 min	3 min	*P* value
*Staphylococcus aureus* (MRSA)±	6.67 × 10^6^ ± 0.02±	1.04 × 10^6^ ± 0.01	.017±	2.03 × 10^7^ ± 0.05±	1. 5 × 10^6^ ± 0.01	<.001
*Staphylococcus epidermidis*±	4.44 × 10^8^ ± 0.05±	2.1 × 10^6^ ± 0.08	<.001±	3.33 × 10^7^ ± 0.015±	2.9 × 10^6^ ± 0.02	<.001
*Enterococcus faecalis*±	1.03 × 10^7^ ± 0.05±	1.61 × 10^6^ ± 0.01	<.001±	2.95 × 10^7^ ± 0.04±	2.48 × 10^6^ ± 0.01	<.001
*Pseudomonas aeruginosa*	-	-	±	6.06 × 10^9^ ± 0.05±	1.69 × 10^9^ ± 0.01	<.001
*Candida albicans*	-	-	±	2.12 × 10^6^ ± 0.01±	1.52 × 10^9^ ± 0.005	<.001

**Table 3:** Antiseptic solutions' effect on the colony-forming unit counts of Gram-positive bacteria.

Bacterial strain	1 min			3 min		
	PI 0.3% H_2_O_2_ 0.5%	PI 5%	*P* value	PI 0.3% H_2_O_2_ 0.5%	PI 5%	*P* value
*Staphylococcus aureus* (MRSA)±	6.67 × 10^6^ ± 0.02±	2.03 × 10^7^ ± 0.05	.018±	1.04 × 10^6^ ± 0.01±	1.5 × 10^6^ ± 0.01	<.001
*Staphylococcus epidermidis*±	4.44 × 10^8^ ± 0.05±	3.33 × 10^7^ ± 0.015	<.001±	2.1 × 10^6^ ± 0.08±	2.9 × 10^6^ ± 0.02	<.001
*Enterococcus faecalis*±	1.03 × 10^7^ ± 0.05±	2.95 × 10^7^ ± 0.04	<.001±	1.61 × 10^6^ ± 0.01±	2.48 × 10^6^ ± 0.01	<.001

## Discussion

Biofilm formation represents a virulence feature that complicates PJIs. The intraoperative use of antiseptic agents is a procedure for preventing surgical site infections. Although the international recommendations advise using 0.3% PI as a prevention tool, highlighting its activity against planktonic bacteria at 1 minute of treatment [24], there is little data available about its antibiofilm activity in literature. Parvin et al. [31] have observed the complete eradication of MRSA, *S. epidermidis* and *P. aeurignosa* mature biofilms (72 hours) after 24 hours of treatment with 10% PI. Gryson et al. [32] observed the 10% PI antibiofilm efficacy on many microbial species at different stages of biofilm maturity (from 2 to 7 days) after 0.5 hours of treatment.

Even though PI solutions are widely used, most surgeons use solutions containing H_2_O_2_. H_2_O_2_, compared to other antimicrobials, has the advantage of producing nontoxic by-products and can potentially help mechanical debridement with its effervescence [33]. Indeed, more recently, Chen et al. showed that H_2_O_2_ lavage before wound closure in multi-segmental lumbar spine surgery could effectively reduce blood loss and surgical site infection rate through an indirect mechanism correlated to its hemostatic properties [34]. Diluted lavage with H_2_O_2_ has shown excellent results in reducing infection rates in primary and revision arthroplasty, especially when mixed with PI [35]. The combination of PI and H_2_O_2_ is demonstrated not to have any reaction, with no gas or precipitate [33]. Ulivieri et al. [25] tested 6.15% PI and 0.2% H_2_O_2_ for irrigating the surgical wounds of patients undergoing spine surgery during 2009. After using the PI and H_2_O_2_ solution, no wound infection occurred in patients operated on during 2009, and no adverse reactions were observed. By contrast, 7 deep infections (1.5%) were recorded in otherwise healthy patients during 2008. Zubko et al. [22] observed a co-inhibitory in vitro effect of the 2 antiseptics when combined (1 mM I + 1.5 mM H_2_O_2_) on planktonic cells of various species of fungi and bacteria.

In our study, monomicrobial biofilms of MRSA, *S. epidermidis*, *E. faecalis*, *P. aeruginosa,* or *C. albicans* were treated with different solutions of PI and H_2_O_2_, alone and in combination, for 1 and 3 minutes of exposure. These treatment times were chosen as they are compatible with irrigation procedures in orthopaedic surgery.

All solutions were first used against 2-day-old biofilms, and their activity was tested by an evaluation of biofilm metabolic activity. Considering the test strains overall, 5% PI solution was the most active already after 1 minute of exposure. After 3 minutes of exposure, 5% PI produced no significant differences in biofilm metabolic activity compared to the shorter treatment, except for *P. aeruginosa*. The 0.3% PI solution showed an effect comparable to the 5% PI solution only on *E. faecalis* after 3 minutes of treatment. Our data do not seem to be in agreement with those of O'Donnell et al. [36] They used 0.35% PI for 1 minute on several microorganisms including MRSA, *S. epidermidis,* and *P. aeruginosa* considering a nascent (4-hour-old) and mature (72-hour-old) biofilm. The Authors reported a reduction in CFU of at least 92.5% for nascent biofilm and at least 71.9% for mature biofilm. From the analysis of our results, it is evident that 0.3% PI after 1 minute did not have significant antibiofilm activity against *S. epidermidis* and *P. aeruginosa*, although in our study the biofilm was less mature (48 hours) compared to the O'Donnell study (72 hours).

Regarding H_2_O_2_ solutions, the antibiofilm activity was always lower than 5% PI except for MRSA biofilm treated for 3 minutes at the highest concentration. Parker et al. [37] evaluated the effect of 24-hour exposure to 0.3% PI or 1.5% H_2_O_2_ against 48-hour-old biofilm of *S. aureus* and reported comparable activity of the 2 solutions (2.2 log reduction in CFU). Our data are not comparable because the experimental conditions are very different, but in our study, the activity of 0.3% PI was significantly different compared to 1.5% H_2_O_2_ solution. It may be that the effects of 0.3% PI and 1.5% H_2_O_2_ become comparable for much longer exposure times. In any case, the exposure time considered by the authors is not compatible with surgical procedures. Presterl et al. [38] evaluated the activity of 10% PI and H_2_O_2_ at different concentrations on 24-hour-old *S.* epidermidis biofilm considering various exposure times. They showed that 0.5% H_2_O_2_ was not bactericidal at all, even at longer exposure times. In our study, 0.5% H_2_O_2_ had no significant effect on the metabolic activity of *S. epidermidis* biofilm at either treatment time. However, based on their data, Presterl et al. concluded that H_2_O_2_ at concentrations of 3% and 5% was more effective than 10% PI in eradicating *S. epidermidis* biofilms. In our study, the antibiofilm activity of 5% PI was significantly greater than that of 1.5% H_2_O_2_, but differences between the 2 studies (biofilm growth age, concentration of the solutions, methods of evaluating the antibiofilm activity) must be taken into account.

The 0.3% PI + 0.5% H_2_O_2_ combined solution was overall less effective than 5% PI. However, considering only the Gram-positive strains, it showed an antibiofilm activity comparable to 5% PI after 3 minutes of exposure. The effect of 5% PI + 1.5% H_2_O_2_ solution did not differ significantly compared to 5% PI at both treatment times, except for *P. aeruginosa* biofilm. Thus, the 5% PI + 1.5% H_2_O_2_ combined solution did not increase the activity of 5% PI alone, while a lower concentration of PI (0.3% PI) in combination with 0.5% H_2_O_2_ allowed to obtain effects comparable to 5% PI on Gram-positive biofilm. Premkumar et al. [39] described the antibiofilm activity of a combined solution (10% PI + 4% H_2_O_2_) against methicillin-sensitive Staphylococcus aureus on different surfaces of common implants and observed an almost total eradication rate after 3 minutes of treatment without significant differences compared to 10% PI alone.

Clinical biofilms can have a highly variable formation age. Biofilm growth age influences its structure and the metabolic state of biofilm embedded cells; mature biofilms are known to be highly recalcitrant to antibiotic treatment and host immune responses. Consequently, the 5% PI and 0.3% PI + 0.5% H_2_O_2_ solutions were evaluated on 5-day-old biofilms, as a 2-day biofilm could be a model very far from the clinical situation. The antibiofilm effect of the 2 solutions was evaluated both by measuring metabolic activity and by counting CFU. The 5% PI solution was tested on mature biofilms of all test strains, while the 0.3% PI + 0.5% H_2_O_2_ solution was evaluated only on mature biofilms of Gram-positive strains. The 5% PI solution caused a significant but less notable reduction in the metabolic activity of mature biofilm compared to the 2-day-old biofilm, with no significant differences between the 2 exposure times. Regarding the 0.3% PI + 0.5% H_2_O_2_ combined solution, after 3 minutes of treatment, the reduction in metabolic activity of the mature biofilm of Gram-positive strains was statistically comparable to that on the 2-day biofilm, and therefore significantly greater than that of 5% PI. Regarding the CFU counts, 0.3% PI + 0.5% H_2_O_2_ solution produced a statistically comparable reduction in 5-day biofilm CFU compared to 5% PI after 3 minutes of treatment. In agreement with Premkumar et al. [39], in our study, the test evaluating the biofilm metabolic activity was less sensitive than the CFU count since the percentages of reduction in CFU observed compared to the control were always greater than the reduction in metabolic activity of the same biofilm treated with the same solution.

An essential requirement for the clinical use of antiseptic solutions is the absence of toxicity to soft tissues. In a previous work, we evaluated the cytotoxicity of the solutions used in the present study (0.3% PI, 0.5% PI, 0.5% H_2_O_2_, 1.5% H_2_O_2_) after 1, 3, and 5 minutes of exposure, showing a gradual increase in cytotoxicity depending on the exposure time, except for 0.3% PI, which had the lowest toxicity [40]. Moreover, we have shown that combined solutions have a cytotoxicity not higher than the individual components at the same concentration. Therefore, based on our data, in the case of PJI by Gram-positive bacteria, 0.3% PI + 0.5% H_2_O_2_ could be used for wound irrigation for 3 minutes of exposure, benefiting from the synergistic effect of the combination of the 2 disinfectants and the antihemorrhagic activity of H_2_O_2_. In the case of PJI with a different etiological agent or PJI with an unknown etiology, it is advisable to use 5% PI for 1 minute of exposure. This study presents some limitations. First, since it is an in vitro test, these results are not necessarily applicable in vivo. Possible interactions with the host’s tissues and immune system could significantly change the susceptibility of these microbial species to these solutions. Secondly, we tested the activity of microbial biofilm on a flat-bottomed 96-well microtiter plate, but different surfaces may likely influence biofilm eradication rates. Finally, we have not tested solutions with intermediate concentrations that could present other behaviors in bacterial biofilm. Furthermore, we recognize that biofilm is not the only mechanism promoting microbial persistence in device-associated infections. Nonetheless, the strengths of this study also deserve mention. To the best of our knowledge, this is the first study that considers different solutions of PI and H_2_O_2_, alone or in combination, evaluating their activity on preformed biofilm of a broad spectrum of microorganisms. Unlike previous works that studied the antibiofilm activity of antiseptic compounds considering longer exposure times, we chose 2 exposure times (1 vs 3 minutes) to assess the minimum irrigation time appropriate for intraoperative use.

## Conclusions

The choice of the antiseptic irrigation solution depends on the germ involved in the biofilm-related infection. Therefore, in the case of PJI by Gram-positive bacteria, 0.3% PI + 0.5% H₂O₂ could be used for wound irrigation for 3 minutes of exposure. In the case of PJI with a different etiological agent or PJI with unknown etiology, it is advisable to use 5% PI for 1 minute of exposure.

## Availability of data and material

The datasets generated and/or analyzed during the current study are not publicly available but are available from the corresponding author on reasonable request.

## Conflicts of interest

The authors declare there are no conflicts of interest.

For full disclosure statements refer to https://doi.org/10.1016/j.artd.2024.101468.

## CRediT authorship contribution statement

**Emanuela Roscetto:** Writing – review & editing, Writing – original draft, Supervision, Methodology, Formal analysis, Conceptualization. **Donato Di Gennaro:** Writing – review & editing, Writing – original draft, Visualization, Validation, Software, Methodology, Conceptualization. **Tiziana Ascione:** Supervision, Methodology, Investigation, Conceptualization. **Umberto Galdiero:** Software, Resources, Investigation, Formal analysis, Data curation. **Martina Aversa:** Writing – review & editing, Writing – original draft, Methodology, Investigation. **Enrico Festa:** Writing – original draft, Visualization, Software, Conceptualization. **Maria Rosaria Catania:** Methodology, Writing – review & editing. **Giovanni Balato:** Writing – review & editing, Writing – original draft, Validation, Supervision, Project administration, Methodology, Conceptualization.

## Appendix A Supplementary data

Conflict of Interest Statement for Roscetto, Di Gennaro, Ascione, Galdiero, Festa, Aversa, and Balato

Appendix 1

Conflict of Interest Statement for Catania
