# Supplementary material for: Antiseptics’ Concentration, Combination, and Exposure Time on Bacterial and Fungal Biofilm Eradication
Source: Arthroplast Today. 2024 Jul 23;28:101468. doi: 10.1016/j.artd.2024.101468 (PMC11320471; doi:10.1016/j.artd.2024.101468)
Supplement: Appendix 1 [file mmc2.docx]

Activity of test solutions against *MRSA* 2 days biofilm determined by XTT assay

|  | OD (490 nm) | |  |  |
| --- | --- | --- | --- | --- |
| *S. aureus* | 1 min | 3 min | | p Value |
| PI 0.3%/ H_2_O_2_ 0.5% | 0,61 ± 0,04 | 0,61 ± 0,002 | | p = 0,7 |
| H_2_O_2_ 0.5% | 2,23 ± 0,18 | 1,72 ± 0,04 | | p =0,242 |
| PI 0.3% | 1,62 ± 0,09 | 0,79 ± 0,07 | | p=0,660 |
| PI 5%/ H_2_O_2_ 1.5% | 0,58 ± 0,03 | 0,62 ± 0,06 | | p=0,674 |
| H_2_O_2_ 1.5% | 1,02 ± 0,06 | 1,20 ± 0,15 | | p= 0,485 |
| PI 5% | 0,55 ± 0,05 | 0,59 ± 0,03 | | p=0,15 |
| Control | 2,51 ± 0,08 | 2,52 ± 0,14 | |  |
| p Value | p < 0.01 | p < 0.01 | |  |

Activity of test solutions against *SE* 2 days biofilm determined by XTT assay

|  | OD (490 nm) | |  |
| --- | --- | --- | --- |
| *S. epidermidis* | 1 min | 3 min |  |
| PI 0.3%/ H_2_O_2_ 0.5% | 2,60 ± 0,03 | 0,77 ± 0,03 | p =0,026 |
| H_2_O_2_ 0.5% | 2,66 ± 0,14 | 2,62 ± 0,04 | p =0,138 |
| PI 0.3% | 2,60 ± 0,17 | 1,38 ± 0,31 | p=0,229 |
| PI 5%/ H_2_O_2_ 1.5% | 0,86 ± 0,06 | 0,89 ± 0,09 | p=0,412 |
| H_2_O_2_1.5% | 1,34 ± 0,05 | 0,96 ± 0,01 | p= 0,394 |
| PI 5% | 0,80 ± 0,04 | 0,81 ± 0,04 | p=0,924 |
| Control | 2,67 ± 0,07 | 2,67 ± 0,07 |  |
| p Value | p < 0.01 | p < 0.01 |  |

|  | OD (490 nm) | |  |  |
| --- | --- | --- | --- | --- |
| *E. faecalis* | 1 min | 3 min | | p Value |
| PI 0.3%/ H_2_O_2_ 0.5% | 0,67 ± 0,06 | 0,61 ± 0,04 | | p = 0,895 |
| H_2_O_2_ 0.5% | 1,28 ± 0,07 | 1,16 ± 0,15 | | p = 0,361 |
| PI 0.3% | 0,81 ± 0,05 | 0,73 ± 0,04 | | p= 0,910 |
| PI 5%/ H_2_O_2_ 1.5% | 0,77 ± 0,02 | 0,63 ± 0,03 | | p= 0,912 |
| H_2_O_2_ 1.5% | 1,06 ± 0,06 | 1,02 ± 0,10 | | p= 0,241 |
| PI 5% | 0,73 ± 0,06 | 0,65 ± 0,01 | | p=0,540 |
| Control | 2,56 ± 0,03 | 2,56 ± 0,03 | |  |
| p Value | p < 0.01 | p < 0.01 | |  |

Activity of test solutions against *E. faecalis* 2 days biofilm determined by XTT assay

|  | OD (490 nm) | |  |  |
| --- | --- | --- | --- | --- |
| *P. aeruginosa* | 1 min | 3 min | | p Value |
| PI 0.3%/ H_2_O_2_ 0.5% | 2,48 ± 0,16 | 1,86 ± 0,43 | | p = 0,292 |
| H_2_O_2_0.5% | 2,76 ± 0 ,12 | 2,71 ± 0,09 | | p = 0,045 |
| PI 0.3% | 2,60 ± 0,28 | 2,41 ± 0,14 | | p= 0,449 |
| PI 5%/ H_2_O_2_1.5% | 1,79 ± 0,08 | 1,50 ± 0,20 | | p= 0,083 |
| H_2_O_2_1.5% | 1,63 ± 0,34 | 1,11 ± 0,11 | | p= 0,219 |
| PI 5% | 0,85 ± 0,14 | 0,61 ± 0,03 | | p= 0,041 |
| Control | 2,72 ± 0,07 | 2,72 ± 0,07 | |  |
| p Value | p < 0.01 | p < 0.01 | |  |

Activity of test solutions against *P. aeruginosa* 2 days biofilm determined by XTT assay

|  | OD (490 nm) | |  |
| --- | --- | --- | --- |
| *C. albicans* | 1 min | 3 min |  |
| PI 0.3%/ H_2_O_2_ 0.5% | 1,03 ± 0,02 | 0,76 ± 0,01 | p= 0,041 |
| H_2_O_2_ 0.5% | 1,50 ± 0,05 | 1,30 ± 0,02 | p= 0,20 |
| PI 0.3% | 1,12 ± 0,10 | 0,79 ± 0,02 | p= 0,112 |
| PI 5%/ H_2_O_2_ 1.5% | 0,64 ± 0,03 | 0,52 ± 0,01 | p= 0,225 |
| H_2_O_2_1.5% | 1,07 ± 0,07 | 0,61 ± 0,02 | p= 0,090 |
| PI 5% | 0,59 ± 0,02 | 0,51 ± 0,02 | p= 0,965 |
| Control | 1,73 ± 0,05 | 1,73 ± 0,05 |  |
| p Value | p < 0.01 | p < 0.01 |  |

Activity of test solutions against *C. albicans* 2 days biofilm determined by XTT assay

|  | OD (490 nm) | |  |  |
| --- | --- | --- | --- | --- |
| PI 5% | 1 min | 3 min | | p Value |
| *Staphylococcus aureus* (MRSA) | 1,32 ± 0,004 | 1,27 ± 0,002 | | p < 0.001 |
| *Staphylococcus epidermidis* | 1,52 ± 0,03 | 1,43 ± 0,03 | | p < 0.001 |
| *Enterococcus faecalis* | 1,39 ± 0,08 | 1,32 ± 0,31 | | p< 0.001 |
| *Pseudomonas aeruginosa* | 1,96 ± 0,26 | 1,64 ± 0,61 | | p< 0.001 |
| *Candida albicans* | 1,15 ± 0,35 | 1,20 ± 0,4 | | p< 0.001 |
| P value | P< 0.001 |  | |  |
|  |  |  | |  |

Activity of PI 5% against Bacterial 5 days biofilm determined by XTT assay

|  | OD (490 nm) | |  |  |
| --- | --- | --- | --- | --- |
| PI 0.3%/ H_2_O_2_ 0.5% | 1 min | 3 min | | p Value |
| *Staphylococcus aureus* (MRSA) | 0,88 ± 0,02 | 0,76 ± 0,03 | | p < 0.001 |
| *Staphylococcus epidermidis* | 3,03 ± 0,01 | 0,74 ± 0,02 | | p < 0.001 |
| *Enterococcus faecalis* | 0,99 ± 0,08 | 0,84 ± 0,01 | | p< 0.001 |
| P value | p< 0.001 | P = 0.04 | |  |

Activity of PI 0.3%/ H_2_O_2_ 0.5%against Bacterial 5 days biofilm determined by XTT assay
