# Supplementary material for: Antiseptics’ Concentration, Combination, and Exposure Time on Bacterial and Fungal Biofilm Eradication
Source: Arthroplast Today. 2024 Jul 23;28:101468. doi: 10.1016/j.artd.2024.101468 (PMC11320471; doi:10.1016/j.artd.2024.101468)
Supplement: Conflict of Interest Statement for Catania [file mmc3.pdf]

CONFLICT OF INTEREST STATEMENT

American Association of Hip and Knee Surgeons

(Adopted from the American Academy of Orthopaedic Surgeons disclosure statement)

Manuscript Title Antiseptics'concentration, combination and exposure time on bacterial and fungal biofilm eradication

1. Royalties from a company or supplier: NONE

2. Speakers bureau/paid presentations for a company or supplier: NONE

3A. Paid employee for a company or supplier: NONE

3B. Paid consultant for a company or supplier: NONE

3C. Unpaid consultants for a company or supplier. NONE

4. Stock or stock options in a company or supplier: NONE

5. Research support from a company or supplier as a Principal Investigator: NONE

6. Other financial or material support from a company or supplier: NONE

7. Royalties, financial or material support from publishers: NONE

8. Medical/Orthopaedic publications editorial/governing board: Editorial board member of BMC Infectious Diseases

9. Board member/committee appointments for a society: NONE

<u>Each author must sign AND print or type his/her name, date and submit a separate form</u>

In addition, one BLINDED Conflict of Interest form (no author names used) should be submitted per manuscript with all author disclosures.

CATANIA MARIA ROSARIA	Maria Rosaria Catania	03/07/2024
Author Name (Print or Type)	Author Signature	Date
